# The right ventricle as the Achilles' heel: tricuspid regurgitation and right heart failure in the era of mechanical circulatory support

**DOI:** 10.3389/fcvm.2026.1946476

**Published:** 2026-10-15

**Authors:** Wentao Wu, Shuohao Wang, Gaojun Xu, Fengjuan Liu, Zhenghe Liu, Xuan Wu, Xia Gao, Zhenwei Ge

**Affiliations:** 1 Department of Cardiovascular Surgery, Henan Chest Hospital, Zhengzhou, China; 2 The Chinese University of Hong Kong, Hong Kong, China; 3 Department of Thoracic Surgery, Henan Provincial Chest Hospital, Zhengzhou University, Zhengzhou, China; 4 Department of Medical Oncology, Henan Provincial Chest Hospital, Chest Hospital of Zhengzhou University, Zhengzhou, Henan, China

**Keywords:** atrial functional tricuspid regurgitation, cardiogenic shock, left ventricular assist device, mechanical circulatory support, right ventricular failure, right ventricular–pulmonary arterial coupling, transcatheter tricuspid intervention, tricuspid regurgitation

## Abstract

Across the mechanical circulatory support (MCS) spectrum, from temporary devices in acute cardiogenic shock to durable left ventricular assist devices, the right ventricle (RV) and tricuspid valve are often underestimated determinants of outcome. This narrative review centers on the RV–tricuspid unit across advanced heart failure and cardiogenic shock, with MCS—temporary and durable and left-, right-, or biventricular—as its principal setting; transcatheter tricuspid trials conducted largely outside MCS are used as the best available causal evidence on whether correcting tricuspid regurgitation changes symptoms and events, and are explicitly distinguished from MCS-specific observational data. Three fields have advanced largely in isolation: transcatheter tricuspid intervention, right ventricular failure risk stratification, and mechanical support of the failing right heart. This review synthesizes the pathophysiology of the RV–tricuspid unit and its prognostic role in durable LVAD therapy and acute shock. It appraises the arrival of transcatheter tricuspid repair and replacement and the limits of the supporting evidence. It also confronts three persistent controversies: concomitant tricuspid surgery at LVAD implantation, the split between symptom and survival benefit after tricuspid intervention, and the timing of right ventricular support. Throughout, we distinguish established evidence from reasonable inference and untested hypothesis. We propose a unified, RV-first framework (assess the unit, stage the ventricle, time the intervention) that recasts these controversies as testable hypotheses, and we present its treatment triggers as research propositions for stage-stratified trials rather than as validated clinical recommendations.

## 1 Introduction

Advanced heart failure and cardiogenic shock impose a rising clinical and economic burden. The population supported by mechanical circulatory support (MCS), from temporary devices in acute shock to durable left ventricular assist devices (LVADs), continues to expand. Contemporary durable LVAD therapy now delivers five-year survival above 58% with fully magnetically levitated pumps (1) and the first adequately powered randomized trial of a microaxial flow pump—a left-sided device (Impella CP) in infarct-related shock, from which overt right ventricular failure was excluded— has shown a mortality benefit in infarct-related shock (2). Yet as left ventricular support has matured, a recurring, under-recognized determinant of outcome has emerged on the other side of the septum: the right ventricle.

The right ventricle (RV) is particularly load-sensitive. Its thin-walled, load-sensitive architecture tolerates acute increases in afterload poorly, and its function is inseparable from the tricuspid valve and the pulmonary circulation (3, 4). When RV–pulmonary arterial coupling fails, secondary tricuspid regurgitation (TR), annular dilatation, and systemic venous congestion form a self-reinforcing cycle. That cycle drives cardiorenal and cardiohepatic injury and predicts death independently of left-sided function (5–7). Impaired RV–PA coupling and RV free-wall strain carry prognostic weight across the whole heart failure spectrum (8, 9). Moderate-or-greater TR independently shortens survival in both community and heart-failure populations (10–12). In the MCS setting these vulnerabilities are amplified: RV failure after LVAD implantation affects about a quarter of recipients early and persists as a late phenotype (13, 14) while acute RV failure remains a pivotal—and sometimes decisive—event in cardiogenic shock, whose reported frequency depends on the definition and population studied.

Three fields have advanced in parallel to address this problem, each rapidly but largely in isolation. First, transcatheter tricuspid intervention has moved from proof-of-concept to randomized evidence; tricuspid edge-to-edge repair (15) and, more recently, transcatheter tricuspid valve replacement (16) show that TR can be reduced safely in patients once considered untreatable. Second, the pathophysiology and risk stratification of RV failure, particularly after LVAD implantation, have been refined into validated scores and standardized definitions (17–19). Third, mechanical support of the failing right heart, from percutaneous RV assist devices to the interaction of the RV with veno-arterial extracorporeal support, has produced a growing but still-contested evidence base (20–22).

Despite obvious points of contact, these literatures rarely speak to one another. Decision-making remains left-ventricle-centric and isolated: the timing of tricuspid intervention, the value of concomitant tricuspid surgery at LVAD implantation (23, 24) and the selection of RV support in shock are each debated within their own field, without an integrating framework. The result is that clinicians managing the same organ, the RV–tricuspid unit, apply inconsistent and sometimes contradictory logic depending on which door the patient enters.

In this review we argue that RV–tricuspid dysfunction is the shared, and frequently underestimated, determinant of outcome across the full MCS spectrum (Central Illustration) (Figure 1). The current fragmented approach should give way to a unified, RV-first framework for assessment, staging, and intervention timing. Existing reviews have treated tricuspid intervention, post-LVAD right ventricular failure, and acute right-heart support as separate subjects. Our contribution is to show that they are governed by the same unit and the same timing logic, and to turn that claim into an explicit, cross-setting staging instrument and decision pathway that can be tested and refuted rather than merely asserted. We first synthesize the pathophysiology of the RV–tricuspid unit. We then examine its prognostic role in durable LVAD therapy and in acute shock, review the arrival of transcatheter tricuspid intervention and its limits in the MCS population, and propose the integrated RV-first framework, defining the evidence gaps that ongoing trials such as TRIC-I-HF (25) are positioned to close. Terminology: throughout we distinguish (i) RV dysfunction (an objective reduction in RV contractile performance), (ii) hemodynamic RV failure (inability of the RV to sustain adequate forward flow without elevated filling pressures or inotropic/mechanical support), and (iii) systemic right-heart failure with congestion (the clinical syndrome of systemic venous congestion and end-organ dysfunction); for post-implant events we adopt the MCS-ARC categories and time windows. We use “threshold” to mean the transition to advanced or potentially irreversible right-heart failure—a composite of hemodynamic decompensation, RV–pulmonary arterial uncoupling, emerging end-organ injury and loss of contractile reserve—rather than any single validated numerical cut-point, because no such cut-point currently exists.

**Figure 1:**
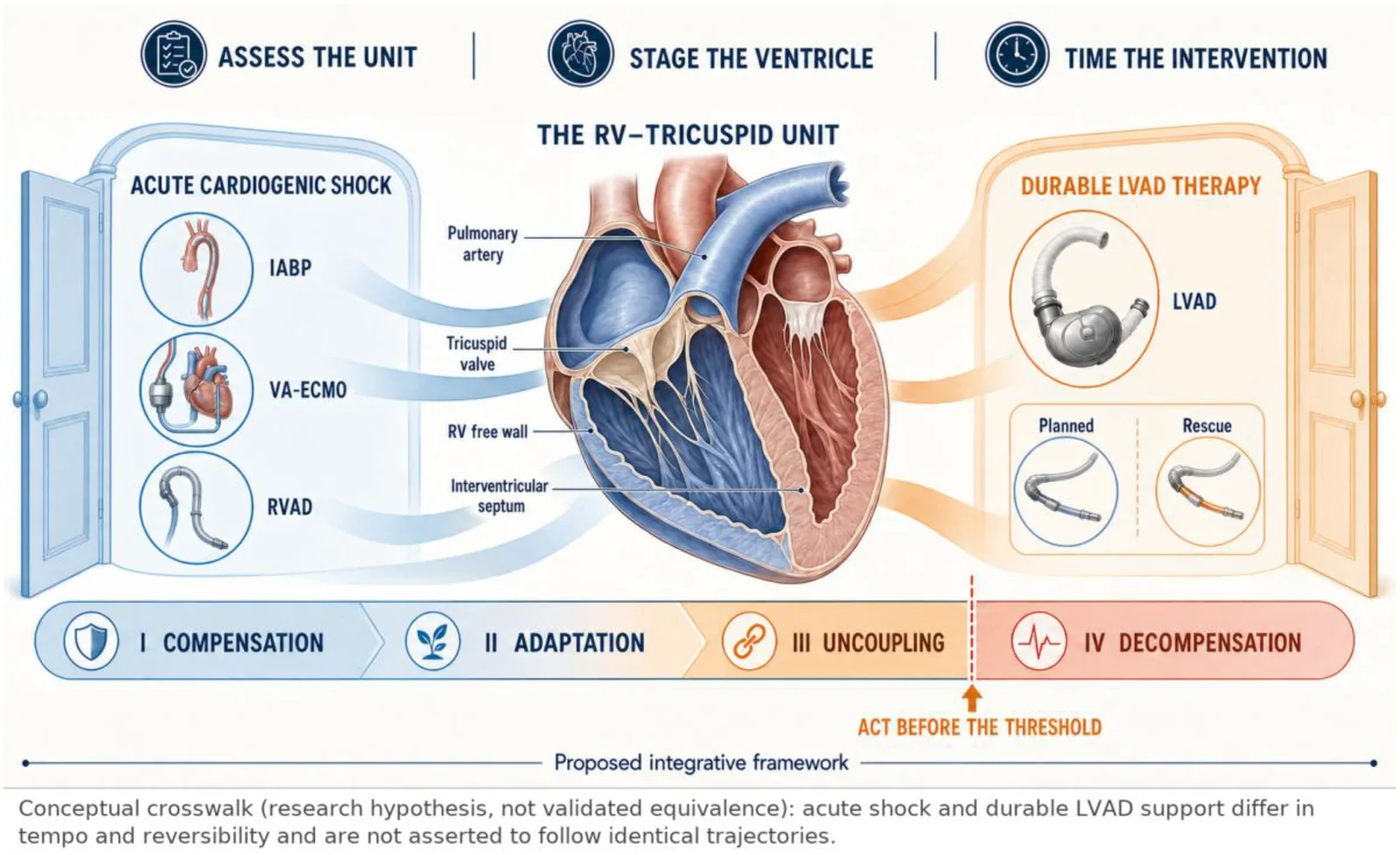
The right ventricle–tricuspid unit as a shared, and frequently underestimated, determinant of outcome across the mechanical circulatory support (MCS) spectrum. A single organ, the coupled right ventricle, tricuspid valve, pulmonary circulation, and interventricular septum, is approached through two doors: acute cardiogenic shock and durable left ventricular assist device therapy. Both share one staging axis (compensation to decompensation) and one timing principle, expressed as three pillars: assess the unit, stage the ventricle, and time the intervention before the prognostic threshold. The two settings differ in tempo and reversibility; the shared I–IV axis is a conceptual crosswalk (research hypothesis), not a validated equivalence between acute and chronic trajectories.

## 2 Materials and methods

### 2.1 Literature search and evidence selection

This is a narrative (non-systematic) review. We searched PubMed/MEDLINE, Embase, the Cochrane CENTRAL register, ClinicalTrials.gov and major society guideline repositories from inception through 12 September 2026 (date of last search); there was no language restriction for core guideline documents, while peer-reviewed English-language literature was prioritized. Principal search terms combined “right ventricular failure” OR “right heart failure” with “mechanical circulatory support”, “left ventricular assist device”, “right ventricular assist device”, “VA-ECMO”, “cardiogenic shock”, “tricuspid regurgitation”, “transcatheter tricuspid valve intervention” and “right ventricular–pulmonary arterial coupling”, together with named trials and registries (MOMENTUM 3, RECOVER RIGHT, IMP-IT, DanGer Shock, TRILUMINATE, TRISCEND, TRIC-I-HF, EUROMACS, STS-INTERMACS, MCS-ARC, SCAI SHOCK and SHARC). Eligible sources comprised randomized and observational studies, meta-analyses, registries, mechanistic and imaging studies, and contemporary guidelines/scientific statements; animal and purely engineering studies were excluded except where they clarified mechanism. Titles/abstracts and full texts were screened and numerical claims and trial outcomes were checked against primary reports. Priority was given to randomized evidence, multicenter registries and current guidelines, followed by high-quality single-center series; study design and level of evidence are stated wherever evidence is cited. Two authors independently verified the numerical claims and citations, with disagreement resolved by consensus. Because the work is conceptual rather than a systematic review, no PRISMA flow diagram or meta-analytic pooling was performed.

## 3 Pathophysiology of the RV–tricuspid unit

The arguments that follow treat the right ventricle, the tricuspid valve, the pulmonary circulation, and the interventricular septum not as separate structures but as a single functional unit. This section establishes that physiology first. The recurring clinical failures examined later (ineffective valve surgery, symptom-only benefit from transcatheter repair, and futile late right ventricular support) are all consequences of treating one component while ignoring the state of the whole.

### 3.1 The right ventricle and its load

The right ventricle is built for a low-impedance circulation. Its thin free wall and crescentic geometry eject large volumes against low pressure efficiently, but cope poorly with acute increases in afterload (4). The relationship between the RV and its load is best expressed as ventriculo-arterial coupling, the ratio of end-systolic to arterial elastance (Ees/Ea). This ratio quantifies how well contractility is matched to the load the RV must overcome (26). Under rising afterload the RV first preserves coupling by raising contractility (homeometric adaptation). As this reserve is exhausted, it dilates to maintain stroke volume (heterometric adaptation), the transition that marks the onset of uncoupling and clinical failure (3). These concepts, derived from invasive pressure–volume analysis, have been validated against cardiac magnetic resonance and, at the bedside, against the TAPSE/PASP ratio, now the most widely used noninvasive surrogate of RV–arterial coupling (27, 28). Impaired coupling, whether measured invasively or by TAPSE/PASP, independently predicts mortality across pulmonary hypertension and the full heart-failure spectrum (8, 29, 30).

### 3.2 Ventricular interdependence

The right and left ventricles are mechanically coupled through the shared interventricular septum and the common pericardium. The loading state of one therefore alters the geometry and function of the other. Acute RV pressure or volume overload displaces the septum leftward and impairs left ventricular filling. This effect was shown directly in humans four decades ago, and it explains why RV failure produces a low-output state even when left ventricular contractility is preserved (4, 31). The interdependence is bidirectional, which creates one of the central paradoxes of mechanical support: unloading the left ventricle can compromise the right. Left ventricular decompression by an LVAD increases venous return to the RV and shifts the septum away from it, reducing the septal contribution to RV ejection on which the failing RV disproportionately depends. This is the mechanistic basis for post-LVAD RV failure examined in Section 4. The converse holds for peripheral veno-arterial extracorporeal support. Its two circulatory effects oppose each other: venous drainage directly lowers RV preload and venous return, whereas retrograde arterial infusion raises left ventricular afterload and, through left ventricular distension and pulmonary congestion, can secondarily increase RV afterload. Net RV loading therefore depends on cannulation configuration, pump flow, residual native cardiac output, pulmonary vascular resistance and ventilation, rather than being uniformly reduced (32). A nominally left-sided intervention can thus reshape right-sided physiology, and vice versa.

### 3.3 Functional tricuspid regurgitation as a marker of the unit

Most TR in the heart-failure and MCS populations is functional (secondary): the leaflets are structurally normal, and regurgitation arises from distortion of the tricuspid apparatus by the ventricle and atrium. In ventricular functional TR, RV dilatation displaces the papillary muscles and tethers the leaflets, a mechanism dissected in patients and in controlled *in vitro* models that isolate the contributions of annular dilatation and papillary-muscle displacement (7, 33). In atrial functional TR, typified by long-standing atrial fibrillation, annular dilatation occurs without leaflet tethering (34). The clinical importance is that functional TR is usually a readout of the RV–atrial-annular complex rather than a primary lesion. This is why abolishing the regurgitation need not reverse the disease, and why recurrent regurgitation after annuloplasty is driven by tethering height and by RV and annular dilatation rather than by the repair itself (35). Ventricular and atrial functional TR are distinct phenotypes rather than interchangeable labels. Ventricular functional TR tracks RV remodeling, septal geometry and pulmonary pressures, whereas atrial (“atrial secondary”) TR reflects tricuspid annular and right-atrial dilation with relatively preserved leaflets and RV, is over-represented in atrial fibrillation and HFpEF, accounts for an estimated 10%–15% of functional TR in contemporary series, and may regress with rhythm or annular-directed therapy (34, 36). The framework developed here is built predominantly around the ventricular phenotype and should not be assumed to apply equally to atrial functional TR, in which severe regurgitation can coexist with a well-preserved RV.

### 3.4 The congestion cascade and its systemic consequences

Once the RV uncouples and the tricuspid annulus dilates, the resulting regurgitation and elevated right atrial pressure are transmitted backward into the systemic venous circulation. Elevated central venous pressure, not low cardiac output, is the principal driver of worsening renal function in decompensated heart failure. It also independently predicts renal dysfunction and death across a broad range of cardiovascular disease (6, 37). The same congestion injures the liver and the gut, producing the multi-organ dysfunction that makes late intervention futile. The cascade is self-reinforcing (Figure 2): RV dysfunction begets annular dilatation and TR, TR increases volume load and further dilates the RV, and systemic congestion degrades the end organs whose function decides whether any intervention can still succeed (7, 38).

**Figure 2:**
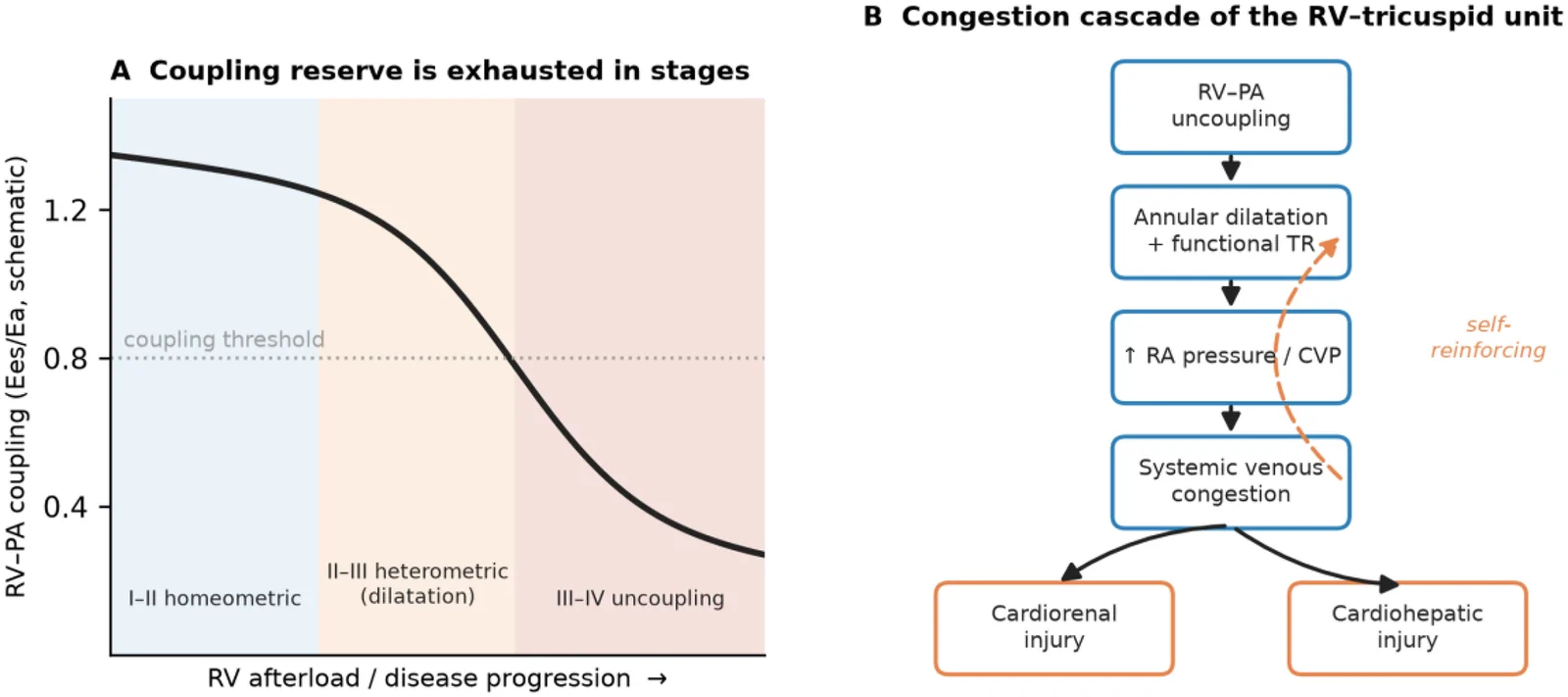
Pathophysiology of the RV–tricuspid unit. **(A)** Right ventricular–pulmonary arterial coupling (Ees/Ea, schematic) is preserved by homeometric adaptation, declines with heterometric dilatation, and falls steeply at uncoupling. **(B)** The congestion cascade: uncoupling drives annular dilatation and functional tricuspid regurgitation, which raise right atrial pressure and central venous pressure, producing systemic venous congestion with cardiorenal and cardiohepatic injury in a self-reinforcing loop.

### 3.5 Why the unit must be assessed as a whole

The consequence is that no single component adequately reports the state of the system. TR grade reflects the valve but not the ventricle; ejection-based indices such as TAPSE are load-dependent and insensitive to early uncoupling; and a normal cardiac output can coexist with advanced congestion. The measures that best capture the unit integrate contractility with load and its downstream effects: RV free-wall longitudinal strain, the TAPSE/PASP coupling surrogate, and staged assessment of venous congestion and end-organ dysfunction. RV free-wall strain is prognostic in heart failure and in significant functional TR, and RV dysfunction is similarly prognostic in HFpEF (9, 39, 40). These are the instruments the RV-first framework (Section 6) draws upon, and the physiology summarized here is why a valve-centric or output-centric assessment repeatedly proves insufficient. Because RV output, geometry and coupling are intrinsically load-dependent, any single resting measurement can misclassify reserve, which motivates the dynamic, serial reassessment developed in Section 6.3 (41, 42). Quantifying this complex, crescentic, retrosternal chamber is itself technically demanding: two-dimensional indices such as TAPSE, fractional area change and longitudinal strain rest on geometric assumptions and dedicated acoustic windows, are angle- and load-dependent, and vary with acquisition and reader (43). Three-dimensional echocardiography (3DE) measures RV volumes and ejection fraction by disk summation without geometric assumptions and quantifies tricuspid annular dimensions and leaflet coaptation, and it compares favorably with cardiac magnetic resonance (CMR), the reference standard for RV volumes, ejection fraction and myocardial tissue characterization; 3DE nevertheless remains constrained by acoustic windows, an enlarged RV, frame rate and feasibility, and—like all RV measures—by loading conditions (43, 44). CMR is preferable when acoustic windows are poor or tissue characterization is required, and concordance across modalities is more reliable than any single measurement (43, 44).

## 4 TR and right heart failure as outcome determinants across the MCS spectrum

Across the mechanical circulatory support (MCS) continuum, right ventricular function is repeatedly implicated as an important determinant of outcome. The presenting problem may be chronic advanced heart failure requiring durable support, or acute infarct-related shock requiring rescue. In either case, the capacity of the RV–tricuspid unit to accommodate an abruptly altered load is associated with both early survival and later trajectory. This section examines that role at the two poles of the spectrum, durable LVAD therapy and acute cardiogenic shock. It shows that the underlying vulnerability, and the controversies it generates, are versions of the same problem.

### 4.1 Right ventricular failure after durable LVAD implantation

Left ventricular unloading by a continuous-flow LVAD does not relieve the right ventricle; it challenges it. Decompression of the left ventricle increases venous return and shifts the interventricular septum leftward. This reduces the septal contribution to RV ejection on which the failing RV depends (4, 31). The clinical consequence, right ventricular failure (RVF), remains one of the principal early complications of durable support, whose reported incidence varies with the definition used. In the standardized STS INTERMACS experience, early RVF affected about one quarter of recipients. A persistent late phenotype was still evident at twelve months in a smaller but prognostically important subset (13, 14). Late RVF, in particular, is an under-recognized driver of readmission, worsening functional status, and death in patients otherwise doing well on left-sided support (14). For consistency we use the MCS-ARC definitions: early acute RV failure (need for RV support at implantation); early post-implant RV failure (RV support within 30 days, inability to wean from inotropes/inhaled nitric oxide within 14 days, or death within 14 days while receiving such support); and late RV failure (RV support after 30 days, or hospitalization after 30 days requiring intravenous diuretics/inotropes for at least 72 h) (17). These time-bounded categories are adverse-event definitions and are distinct from the chronic RV–tricuspid staging used elsewhere in this review.

Anticipating who will develop RVF has occupied the field for more than a decade. The earliest risk models combined markers of end-organ congestion and hemodynamic severity (Table 1). They included the Michigan RV Failure Risk Score (18) and the Penn score for biventricular support (45) later joined by the Utah model (46). In the continuous-flow era, the EUROMACS Right-Sided Heart Failure Risk Score identified independent predictors: the ratio of right atrial to pulmonary capillary wedge pressure (RA/PCWP), hemoglobin, multiple inotrope use, INTERMACS profile, and severe RV dysfunction (19). The pulmonary artery pulsatility index (PAPI), originally derived in acute inferior infarction (47) predicts post-LVAD RVF at a single-center-derived threshold near 1.85, though external validation across cohorts has been inconsistent (48). The modest and inconsistent discrimination of these scores points to a deeper truth. RVF is not a single event set off by a single threshold. It is the failure of an integrated unit whose reserve depends at once on afterload, contractility, rhythm, and valvular competence. Afterload here is not captured by pulmonary pressures alone. Elevated pulmonary vascular resistance, a combined pre- and post-capillary pattern, and the degree to which resistance is fixed rather than reversible (49) all shape whether the RV will tolerate LVAD unloading, and durable support can itself lower an initially high resistance over time.

**Table 1:** Risk and staging tools for the RV–tricuspid unit across MCS settings.

Tool	Class	Setting	Key variables	Predicts/describes	Limitation	Ref
Michigan RV Failure Risk Score	Predictive risk score	Pre-LVAD	Inotrope need, reoperation, low cardiac index, end-organ markers (bilirubin/creatinine/AST)	Post-LVAD RVF	Weak discrimination in continuous-flow era	(18)
Penn score	Predictive risk score	Pre-biventricular	Hemodynamic + end-organ markers	Need for biventricular support	Small derivation cohort	(39)
Utah model	Predictive risk score	Pre-LVAD	Destination therapy, hemodynamic/laboratory markers	Post-LVAD RVF	Limited external validation	(40)
EUROMACS-RHF score	Predictive risk score	Pre-continuous-flow LVAD	RA/PCWP, hemoglobin, multiple inotropes, INTERMACS profile, severe RV dysfunction	Early RVF after CF-LVAD	Modest discrimination across cohorts	(19)
PAPI	Physiological index (load-dependent)	Acute MI/pre-LVAD	(PA systolic−PA diastolic)/RA pressure	Severe RV dysfunction; post-LVAD RVF (commonly cited ∼1.85, cohort-dependent)	Single resting value; threshold varies; inconsistent validation	(41, 42)
TAPSE/PASP	Physiological index (coupling)	HF/PH/TR	Echocardiographic coupling surrogate	RV–arterial uncoupling; mortality	Falls late when TAPSE and PASP drop together	(8, 28)
RV free-wall longitudinal strain	Physiological index	HF/significant functional TR	Speckle-tracking strain	Outcome in HF and significant TR	Vendor/measurement variability	(9, 37)
STS-INTERMACS/MCS-ARC	Adverse-event definition	Post-LVAD	Standardized adverse-event definitions with time windows	Early acute, early post-implant and late RVF	Definitional, not predictive	(13, 17)
SCAI SHOCK stages	Staging system	Acute cardiogenic shock	Clinical/hemodynamic stages A–E	Mortality in cardiogenic shock	Not RV-specific	(76, 77)
Secondary TR/RHF staging	Staging system	Chronic TR	Congestion and end-organ dysfunction with TR grade	Outcome in secondary TR and RHF	Emerging; not cross-setting validated	(36, 75)
3D echocardiography; cardiac magnetic resonance (CMR)	Volumetric & tissue imaging	All settings	3DE: RV volumes, RVEF by disk summation, annular/coaptation dimensions; CMR: reference-standard volumes, RVEF, tissue characterization	RV size/function and valve anatomy	3DE limited by acoustic window/enlarged RV; CMR less available at bedside; both load-sensitive	(90, 91)

Classes are distinct and not interchangeable: predictive risk scores estimate event probability in a defined population; physiological indices characterize current RV state and are load-dependent; staging systems describe clinical trajectory; adverse-event definitions standardize reporting (we use MCS-ARC time windows).

RVF, right ventricular failure; RV, right ventricle; LVAD, left ventricular assist device; CF, continuous-flow; PAPI, pulmonary artery pulsatility index; TAPSE, tricuspid annular plane systolic excursion; PASP, pulmonary artery systolic pressure; RA/PCWP, right-atrial-to-pulmonary-capillary-wedge-pressure ratio; MCS-ARC, mechanical circulatory support academic research consortium; SCAI, society for cardiovascular angiography and interventions; RHF, right heart failure; TR, tricuspid regurgitation; PA, pulmonary artery; MI, myocardial infarction; HF, heart failure; PH, pulmonary hypertension.

Several MCS-specific modifiers cut across this biology and deserve explicit emphasis. In continuous-flow LVADs, pump speed alters interventricular septal position and thus RV geometry and tricuspid coaptation: excessive speed can shift the septum leftward and enlarge the tricuspid annulus, whereas suboptimal speed leaves systemic output dependent on a stressed RV. The supported circulation is strongly preload-dependent, so intravascular volume, the right-atrial-to-pump gradient and fixed vs. reversible pulmonary vascular resistance jointly determine RV output. Atrial or ventricular arrhythmias and loss of atrioventricular synchrony acutely reduce RV contribution; positive-pressure ventilation and high intrathoracic pressure lower venous return and can unmask RV failure after weaning from cardiopulmonary bypass; and perioperative volume, blood-product and inotrope management change the load against which candidacy is judged. In temporary support, the drainage/return configuration (peripheral vs. central; isolated RV vs. combined veno-arterial or biventricular circuits) determines which chamber is unloaded and which is loaded. These factors explain why a static, off-support measurement rarely captures operative RV reserve and why reassessment after optimization is essential (4, 17, 31, 32, 41).

This tension is evident in the enduring controversy over concomitant tricuspid valve surgery at LVAD implantation. The rationale is intuitive, since significant TR compounds venous congestion and might accelerate RVF. Yet the evidence has consistently failed to support the intervention. The pivotal STS database analysis of more than two thousand patients with moderate-or-greater TR found that adding a tricuspid procedure neither reduced the need for RV assist device (RVAD) support nor lowered early mortality, while it increased postoperative morbidity (23). Propensity-matched EUROMACS data reached the same conclusion (24). These are observational studies—a national database and a propensity-matched registry analysis—and no dedicated randomized trial exists; a comprehensive analysis of the >2,200-patient MOMENTUM 3 trial portfolio likewise associated concurrent valve procedures with more RV failure, bleeding and stroke but no survival advantage (50). Registry analyses and single-center series have largely echoed this, reporting limited durability of repair and high rates of recurrent TR (51–53). A minority describe favorable right heart failure trajectories when repair follows strict guideline indication (54). This registry evidence is itself confounded by indication, because sicker patients with greater TR and RV dysfunction were preferentially selected. The apparent absence of benefit therefore cannot be cleanly separated from baseline severity, and genuine equipoise persists for severe TR with annular dilatation. The literature is also heterogeneous in baseline TR severity, repair technique, residual/recurrent TR, device era, RV function and definition of post-operative RV failure; the defensible reading is therefore not that surgery has been shown to “fail,” but that routine, anatomy-triggered intervention has not demonstrated consistent benefit and may add morbidity, whereas benefit in carefully defined, reserve-selected subgroups remains uncertain (50). What the data establish is narrower but instructive. Addressing the valve in isolation, triggered by anatomy rather than ventricular reserve, has not changed the natural history. The same theme reappears with transcatheter therapy (Section 5).

When RVF escalates to the need for mechanical RV support, the timing of that decision appears to carry prognostic weight, though the evidence is observational and must be read with caution. Delayed, unplanned conversion to RVAD support after LVAD implantation carries a poor prognosis: in the Columbia series, patients unable to wean from temporary RV support had 74% in-hospital mortality and only 13% six-month survival (55). Outcomes are markedly better when biventricular support is planned from the outset. This comparison is confounded by indication. Patients requiring rescue conversion are, by definition, those who acutely decompensated, so the difference conflates decision timing with baseline salvageability. It cannot alone establish that earlier action is causally protective. We therefore treat the planned-vs.-rescue gradient as a hypothesis that motivates this review, not a settled conclusion. As the next section shows, that gradient recurs at the acute end of the spectrum.

### 4.2 Acute cardiogenic shock and temporary or right-sided mechanical support

At the acute end of the spectrum, right ventricular function is an important determinant of early survival in cardiogenic shock. Acute RV failure, whether from infarction, pulmonary embolism, or a complication of left-sided support, produces a low-output state. This state is highly sensitive to loading and tolerates poorly the volume and vasopressors that follow by reflex (6, 37). Its hemodynamic signature is captured by the same indices that predict RVF after LVAD: a rising right atrial pressure relative to filling, and a falling PAPI (47).

For years, mechanical support in infarct-related shock accumulated a strikingly negative evidence base. Intra-aortic balloon counterpulsation showed no mortality benefit (56). Randomized trials of veno-arterial extracorporeal membrane oxygenation (VA-ECMO), together with their individual-patient-data meta-analysis, likewise failed to show a survival benefit from routine early support, at the cost of more bleeding and vascular complications; the neutral signal persisted at one year (21, 57–60). Against this backdrop, DanGer Shock marked a turning point. A microaxial flow pump (Impella CP) in STEMI-related shock reduced 180-day all-cause mortality, with an absolute reduction of about 13% (from 59% to 46%) (hazard ratio ≈0.74), albeit with more bleeding, limb ischemia, and renal-replacement therapy. Notably, the trial excluded mechanical complications and comatose out-of-hospital arrest, exclusions that are themselves a form of selection (2). Because DanGer Shock tested left ventricular unloading and excluded overt right ventricular failure, it is not a trial of dedicated RV support or of RV-stage-triggered deployment; its result cannot be extrapolated to earlier RVAD placement or to mixed/biventricular shock. A broader meta-analysis of temporary MCS then localized any benefit to selected patients, those with STEMI and without established hypoxic brain injury, rather than to early, indiscriminate support (61). The consistent message is that device efficacy is inseparable from patient selection, and the state of the RV is a central axis of that selection.

One consideration specific to veno-arterial extracorporeal support deserves emphasis, because it is where left- and right-heart physiology collide. Peripheral VA-ECMO combines two opposing effects: venous drainage directly lowers RV preload and venous return, whereas retrograde arterial flow raises left ventricular afterload and can precipitate LV distension and pulmonary edema, which in turn elevates RV afterload through pulmonary congestion, so the net effect on RV loading is flow- and configuration-dependent (32). Strategies to unload the left ventricle, such as concomitant Impella (ECpella/ECMELLA) (62) intra-aortic balloon counterpulsation, or atrial septostomy, are therefore not merely left-sided concerns. They also determine the load the RV must overcome, and RV recovery frequently governs whether a patient can be weaned from support. This interdependence, introduced physiologically in Section 3.2, means the RV cannot be assessed or supported in isolation, even when the presenting device is nominally left-sided.

A related intervention with an instructive RV-first physiology is the intra-atrial shunt. In cardiogenic shock, or during peripheral VA-ECMO complicated by left-sided distension, percutaneous balloon atrial septostomy can decompress the left heart by venting left atrial pressure, but the resulting left-to-right passage of blood adds volume to the right heart and pulmonary circulation and is meaningful only if the RV can accept the additional load. In chronic heart failure, passive interatrial shunt devices create a controlled left-to-right shunt to lower left atrial pressure, particularly during exercise. The sham-controlled REDUCE LAP-HF II trial was neutral overall (win ratio 1.0 in the primary analysis and 1.01 at two years), with worse outcomes in patients with elevated exercise pulmonary artery pressure or an enlarged right atrium; two-year data showed increases in right ventricular and right atrial volumes (an RV-volume increase of at least 30% in 39% of device recipients vs. 28% of sham participants, *P* < 0.001) without change in RV systolic function, and any signal of benefit was confined to a “responder” phenotype without latent pulmonary vascular disease (63, 64). These findings exemplify the central thesis of this review: an intervention designed to unload the left chamber imposes an additional volume load on the RV, so pulmonary vascular reserve and RV capacity—not left-sided pressures alone—should govern its use, and established RV failure or severe pulmonary vascular disease argues against it (63, 64).

This is the context in which dedicated RV support has developed. The RECOVER RIGHT study established that a percutaneous microaxial RVAD could be deployed safely in medically refractory RVF, including RVF complicating LVAD implantation, with immediate hemodynamic benefit (20). Contemporary devices have broadened the toolkit: the dual-lumen right-atrium-to-pulmonary-artery ProtekDuo cannula, deliverable through a single venous access (65) and the jugular-deliverable Impella RP Flex (66). Real-world experience, however, has been discouraging. Registry cohorts of the Impella RP report in-hospital mortality near 47%, and a large contemporary analysis of temporary percutaneous RVADs found they were used in only a small fraction of shock patients and carried in-hospital mortality above 50% (22, 67). These figures are most plausibly explained, in part, by late deployment into established multi-organ failure rather than by device inadequacy. As unadjusted registry data, though, they cannot exclude that such patients were unsalvageable regardless of timing. No randomized trial has yet defined the optimal timing or device for acute RV support, and this remains one of the field's most consequential evidence gaps.

### 4.3 One organ, two doors

Viewed together, the two poles of the MCS spectrum describe a single pathophysiological entity approached through different doors. In durable support, the RV–tricuspid unit determines whether left-sided unloading succeeds. In acute shock, it determines whether the patient survives to definitive therapy. In both, the prognostic indices are shared (RA/PCWP, PAPI, RV strain), and the dilemma is identical: treat the valve, support the ventricle, or both, and when. The available data are compatible with—but do not establish—the hypothesis that outcome hinges less on the availability of intervention than on its timing, before decompensation becomes irreversible, because earlier treatment is confounded with greater salvageability; stage-stratified trials are required to confirm it. This convergence motivates a unified framework. It is also the prognostic backdrop against which the arrival of transcatheter tricuspid intervention, the subject of the next section, must be judged.

## 5 The arrival of transcatheter tricuspid intervention

For decades the tricuspid valve was the “forgotten valve,” and its neglect was rational in a narrow sense. Isolated tricuspid surgery carried very high operative mortality, much of it attributable to the very right heart failure and end-organ congestion that late referral had allowed to develop (68). Patients were therefore referred late or not at all. The natural history of untreated significant TR, namely progressive venous congestion, cardiorenal and cardiohepatic injury, and excess mortality independent of left-sided disease, was left to run its course (10, 12). Transcatheter tricuspid intervention emerged to break this deadlock by offering valve repair or replacement at a fraction of the surgical risk. Whether it also breaks the prognostic deadlock is the more difficult question this section addresses.

### 5.1 From single-arm signal to randomized evidence

Tricuspid transcatheter edge-to-edge repair (T-TEER) developed first. The early single-arm TRILUMINATE experience showed that leaflet approximation could reduce TR safely, with sustained one-year reductions and clinical improvement in a high-surgical-risk population (69, 70). Registry data broadened the evidence beyond the trial population. The bRIGHT post-approval study, which enrolled a sicker real-world cohort, confirmed high implant success and durable TR reduction, and linked residual TR severity to survival (71, 72). The TriValve registry reinforced these observations: transcatheter intervention was associated with lower one-year mortality and heart-failure hospitalization than propensity-matched medical controls (73). This early comparative signal must be read with caution. Treated patients were, by definition, judged suitable candidates, so residual confounding by candidacy cannot be excluded. As discussed below, the survival signal was not reproduced against a randomized comparator.

The pivotal TRILUMINATE randomized trial then delivered a more nuanced verdict. T-TEER produced a large and durable reduction in TR and a significant improvement in quality of life, meeting a hierarchical composite primary endpoint (win ratio 1.48; *P* = 0.02) to which quality-of-life and functional ranks contributed substantially, whereas the individual death and heart-failure-hospitalization components did not differ significantly and the trial was not powered for mortality. It did not, however, reduce all-cause mortality within the trial's follow-up. A two-year recurrent-event analysis showed fewer heart-failure hospitalizations, yet the demonstrated benefit remained anchored predominantly in symptoms and TR grade (15, 74). This is an absence of demonstrated mortality benefit over the available follow-up, not evidence that mortality is unaffected. This dissociation, unambiguous mechanical and symptomatic success without a demonstrated survival benefit, has become the defining interpretive challenge of the field.

Transcatheter tricuspid valve replacement (TTVR) followed a parallel trajectory, but with more complete TR elimination. Early feasibility and first-in-human EVOQUE experience showed that orthotopic replacement could abolish TR in nearly all treated patients, with durable results and hemodynamic improvement at one year (75–77). The randomized TRISCEND II trial confirmed that TTVR reduced TR to mild or less in the large majority and met its hierarchical composite primary endpoint (win ratio 2.02; *P* < 0.001), which ranked death or RV-support/transplant, subsequent tricuspid intervention, heart-failure hospitalization, quality of life, NYHA class and walk distance; the win was driven principally by the quality-of-life and functional ranks, and the study was not powered for mortality. The individual components of mortality and heart-failure hospitalization were not, on their own, significantly reduced over the follow-up reported (16, 78). Alternative device concepts, including annuloplasty systems and geometry-independent replacement designs, have widened the technical repertoire and are summarized in current guideline and consensus documents (79–82).

Most recently, the investigator-initiated, open-label TRIC-I-HF trial enrolled 360 patients with severe tricuspid regurgitation and overt right-sided heart failure at 29 high-volume German centers, randomized 2:1 to a device-agnostic transcatheter repair strategy (predominantly T-TEER with the PASCAL or TriClip systems) plus medical therapy vs. medical therapy alone. The cohort was high-risk and congested (mean age approximately 80 years; roughly three-quarters in NYHA class III–IV; most with cardiorenal or cardiohepatic syndromes). Both hierarchical primary endpoints were met: at one year the win ratio for the ranked composite of death, heart-failure hospitalization and quality of life was 2.42 (95% CI: 1.76–3.33; *P* < 0.001), and through three years freedom from all-cause death or heart-failure hospitalization was 52.4% vs. 21.0% (hazard ratio 0.40, 95% CI: 0.29–0.55; *P* < 0.001); heart-failure hospitalization was reduced (hazard ratio 0.35, 95% CI: 0.25–0.51), and all-cause survival was higher as an unadjusted secondary endpoint (72.3% vs. 53.9% at three years; hazard ratio for death 0.62, 95% CI: 0.40–0.96) (25, 83). This is the first adequately powered randomized strategy trial to demonstrate reduction in hard clinical events with tricuspid repair. Several caveats apply: the trial was open-label; mortality was a secondary endpoint not adjusted for multiplicity, with three-year follow-up still maturing; and a high crossover rate (43.7% by three years) biases intention-to-treat estimates toward the null. Most relevant to this review, patients were neither stratified nor randomized by RV stage, so TRIC-I-HF establishes event benefit in a selected high-risk cohort without testing whether that benefit depends on ventricular stage or reserve (83).

### 5.2 Why symptom benefit outruns survival benefit

The consistent pattern across T-TEER and TTVR, robust reduction in TR and symptoms but unproven effect on mortality (Figure 3), admits several non-exclusive explanations. Each points back to the right ventricle. First, follow-up in these trials remains short relative to the slow natural history of congestion-driven organ injury, so a survival signal may simply need more time. Second, and more fundamentally, TR in the heart-failure population is frequently the consequence of RV and annular remodeling rather than its cause. Abolishing the regurgitant volume therefore need not reverse the underlying RV dysfunction that drives outcome (7, 38). Third, patient selection may have been suboptimal. Intervening once RV failure is established, analogous to the late-referral problem of the surgical era, treats the valve after the ventricle has already crossed a prognostic threshold. Importantly, limited follow-up, hierarchical estimands, treatment crossover and few events mean that “not demonstrated” is not “demonstrated absent,” and symptom improvement must not be presented as evidence that earlier intervention necessarily prevents death.

**Figure 3:**
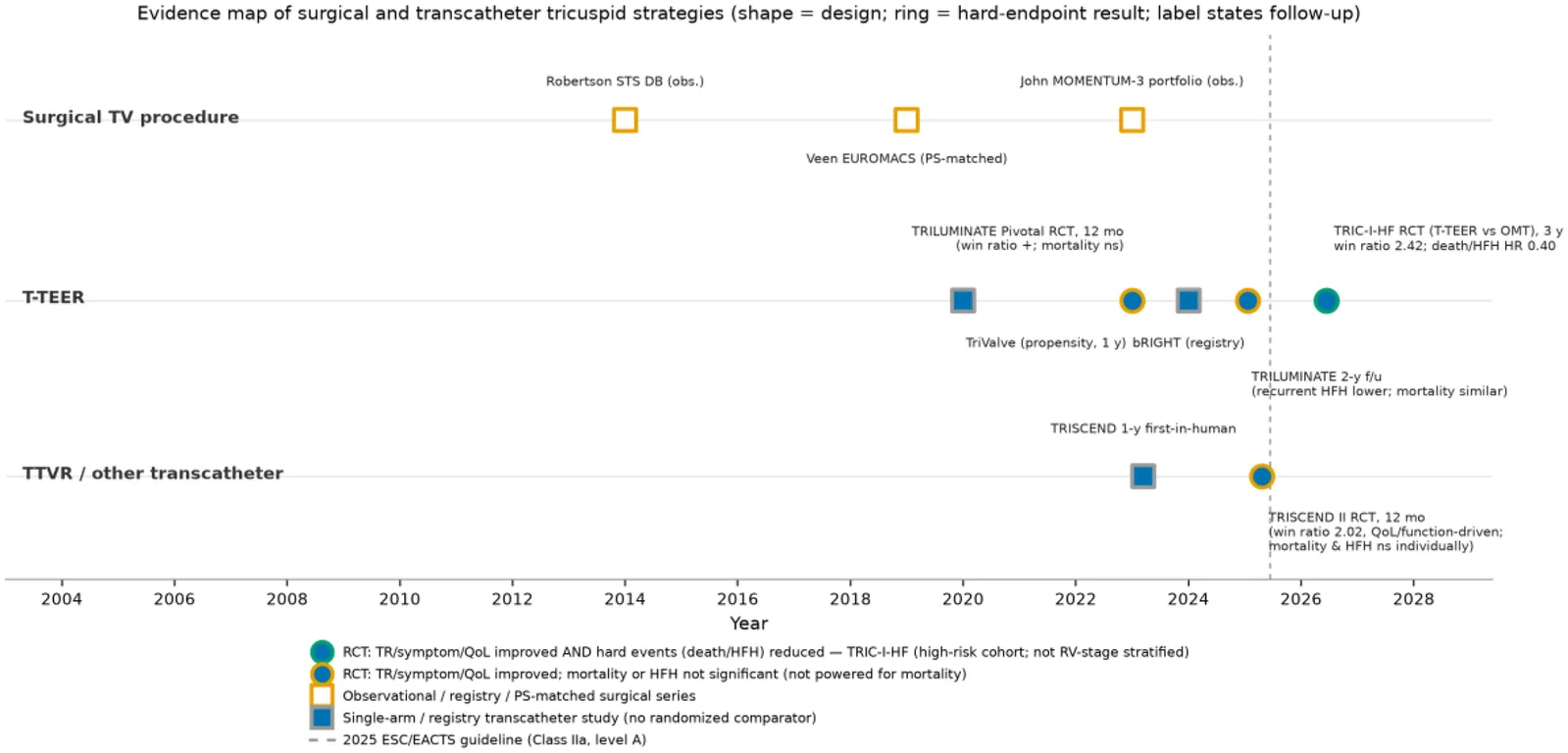
Evidence map of surgical and transcatheter tricuspid strategies over time. Marker shape encodes study design (circle, randomized trial; square, observational/registry/single-arm study); blue fill marks improvement in tricuspid-regurgitation/symptom/quality-of-life endpoints, an orange ring marks non-significant mortality or heart-failure-hospitalization endpoints (pivotal trials were not powered for mortality), a green ring marks a randomized strategy trial that also reduced the hard composite of death or heart-failure hospitalization (TRIC-I-HF, high-risk cohort; not stratified by RV stage), and a grey ring marks absence of a randomized comparator. A color-blind-safe palette is used and each label states design and follow-up; “not demonstrated” is distinct from “demonstrated absent.”.

A clinically relevant consideration is supported, indirectly, by hypothesis-generating evidence. In a multicenter propensity-matched analysis, the survival benefit of transcatheter tricuspid intervention appeared concentrated in patients with mid-range RV function and attenuated in those with advanced RV dysfunction, precisely the gradient a valve-centric selection strategy would miss (84). As a non-randomized subgroup analysis, this is vulnerable to residual confounding and effect-modification artifact and needs prospective confirmation. Even so, together with the physiology, it points toward RV function as the axis along which candidacy and timing should be defined. A complementary mechanistic consideration comes from the procedure itself. Abruptly abolishing severe TR removes a low-pressure “pop-off” for the failing RV and can precipitate acute RV decompensation. Knowing RV reserve before eliminating the regurgitation is therefore particularly important. The prognostic tools developed in the heart-failure and imaging literature are the natural instruments for that selection: RV free-wall strain, TAPSE/PASP coupling (which fails when TAPSE and PASP fall together in advanced disease), and staged assessment of venous congestion and end-organ dysfunction (8, 9, 38, 39). Yet they are inconsistently applied in current tricuspid practice.

Candidacy for transcatheter tricuspid intervention is multidimensional and device-specific. Anatomical feasibility, assessed by transthoracic/transesophageal echocardiography and, for replacement, computed tomography, includes leaflet length and tethering height, the coaptation gap and its location, anterior–septal jet location, the presence and course of pacemaker/defibrillator leads, annular and septolateral dimensions, and vena-caval and right-heart size and trajectory (36, 85). Physiological suitability must be weighed alongside anatomy: pulmonary hypertension and pulmonary vascular resistance, left-sided valvular disease, right atrial/ventricular size, hepatic and renal dysfunction, and frailty all modify feasibility and outcome. Repair and replacement are not physiologically interchangeable: T-TEER reduces the regurgitant orifice while preserving forward ejection, whereas TTVR abolishes the low-impedance regurgitant “pop-off” more abruptly and can expose a weak RV to effective afterload mismatch and acute decompensation when reserve is poor. Device-specific hazards also differ, including bleeding/vascular injury, leaflet injury and single-leaflet detachment with repair, and conduction disturbance/new pacemaker requirement, device thrombosis with its anticoagulation burden, paravalvular leak and RV decompensation with replacement (16, 85). Selection therefore integrates anatomy, RV–pulmonary arterial coupling, congestion and end-organ reserve, and anticipated device-specific risk, rather than TR grade alone.

### 5.3 The interface with the MCS population

The relevance of transcatheter tricuspid intervention to the MCS spectrum is direct but largely unexplored. In candidates for or recipients of durable LVAD support, significant TR is common. The failure of concomitant surgical repair (Section 4.1) raises a question: might a staged transcatheter approach, timed by RV reserve rather than performed by reflex at implantation, succeed where routine surgery did not? Conversely, severe RV dysfunction that makes a patient a poor candidate for isolated tricuspid intervention may identify precisely those who need a ventricular strategy, such as escalation to biventricular support or reconsideration of candidacy, rather than an isolated valve procedure. No randomized data yet address the tricuspid valve in the LVAD population, and this intersection is one of the clearest gaps the field must close.

Taken together, the arrival of transcatheter tricuspid therapy has made the valve treatable at low procedural risk in appropriately selected, anatomically suitable patients—although anatomical exclusions, procedural complications, device durability and limited long-term outcomes remain—and it has sharpened, rather than resolved, the problem of whom to treat and when. The answer to both, we argue in the next section, requires abandoning a valve-centric or device-centric frame for one organized around the right ventricle itself.

## 6 An integrated RV-first framework

The preceding sections describe a field that has advanced impressively along three separate tracks, yet still lacks a shared organizing principle. Risk scores predict RV failure after LVAD implantation but do not inform the timing of tricuspid intervention. Transcatheter therapy abolishes regurgitation but is selected by valve anatomy rather than by ventricular reserve. Acute RV support is deployed, often too late, without reference to the same hemodynamic indices that govern the chronic setting. We propose that these tracks converge on a single object, the RV–tricuspid unit, and that decision-making should be reorganized around it. The framework has three components: assess the unit, not the valve; stage the ventricle, not the regurgitation; and time the intervention before, not after, the prognostic threshold is crossed.

### 6.1 Assess the unit, not the valve

The first principle is that the relevant clinical entity is the coupled RV–tricuspid–pulmonary system, and it must be characterized as such. TR severity alone is a weak guide to prognosis, because identical regurgitant grades carry very different meaning depending on the state of the ventricle behind them (7, 86). The instruments to do better already exist and are validated across the heart-failure, imaging, and pulmonary-hypertension literatures. RV free-wall longitudinal strain outperforms conventional indices for predicting outcome in heart failure and in significant functional TR (9, 39). The TAPSE/PASP ratio serves as a noninvasive surrogate of RV–arterial coupling, validated against invasive pressure–volume-derived Ees/Ea (3, 8). Hemodynamic markers of the unit under load complete the set: right atrial pressure relative to filling, and PAPI (47, 48). The recurring failure of valve-centric strategies, namely concomitant tricuspid surgery at LVAD implantation and isolated transcatheter intervention in patients with advanced RV dysfunction, is in this light a predictable consequence of assessing the wrong thing. When RV function is measured and used, the signal sharpens, as in the propensity-matched analysis showing benefit concentrated in patients with mid-range RV function (84).

### 6.2 Stage the ventricle, not the regurgitation

The second principle is that the RV–tricuspid unit moves through identifiable stages of adaptation and failure. These stages, not the momentary severity of TR, should structure decisions. The physiology is well described. The RV first compensates for increased afterload by homeometric adaptation, then dilates (heterometric adaptation), and finally uncouples from the pulmonary circulation. At that point venous congestion, tricuspid annular dilatation, and secondary end-organ injury become self-reinforcing (3, 26). Staging schemes that capture this trajectory, by incorporating venous congestion and end-organ dysfunction rather than valve grade alone, already predict outcomes in secondary TR and in right heart failure. Analogous logic underlies the RVF definitions used in the LVAD literature and the SCAI stages used in shock (38, 87, 88). A unified framework would apply one staging language across settings, but because these instruments were developed for different populations, Table 3 is presented strictly as a conceptual crosswalk and research hypothesis, not as a validated unified stage, and the source systems retain their population-specific meanings. A patient's position on the compensation-to-failure continuum, rather than the door through which they entered, would then determine whether the appropriate response is surveillance, valve intervention, ventricular support, or reconsideration of candidacy. Table 3 proposes such a language. It aligns the SCAI shock stages, the INTERMACS/EUROMACS RV-failure definitions, and contemporary TR staging onto a common four-stage axis of the RV–tricuspid unit. We deliberately attach no numerical candidate thresholds: because the source cohorts use incompatible measurements, outcomes and time horizons and RV indices are load-dependent, fixed numeric cut-points would imply a precision the evidence does not support. The descriptors are therefore directional only, and dynamic multivariable reassessment is more defensible than any single resting value; prospective calibration in stage-stratified cohorts is required before any numeric cut-point could be used to select treatment.

### 6.3 Time the intervention before the threshold

The third principle, and the one presented here as a plausible, testable hypothesis rather than an established law, because the supporting data are observational and cannot separate a treatment-timing effect from confounding by indication, immortal-time bias, survivor selection and differences in baseline salvageability, is that timing may be a major determinant of outcome. The same gradient appears in every setting reviewed. In durable support, planned biventricular support is tolerable, while delayed, unplanned RVAD conversion carries 74% in-hospital mortality (and only 13% six-month survival) in patients unable to wean from temporary support (55). In acute shock, dedicated RV support deployed into established multi-organ failure yields registry mortality above 50%, whereas the trials suggesting benefit did so through earlier, selected application; DanGer Shock, however, tested left-sided unloading and excluded overt RV failure, so it is only indirect and is not a test of stage-triggered RV support (2, 22, 61). In transcatheter tricuspid therapy, the dissociation between symptom benefit and survival benefit is most simply explained by intervention after the RV has already crossed a prognostic threshold, a repeat of the surgical era's late-referral problem (15, 84). Across all three, the decisive variable is not whether an intervention is available but whether it is applied while the unit still has reserve. An RV-first framework makes this explicit by defining intervention windows in terms of ventricular stage rather than symptom onset or valve grade. Figure 4 renders this as a two-tier decision pathway that acknowledges the different tempos of the two doors. The first is a rapid bedside hemodynamic tier for acute shock, where strain and cross-sectional imaging are often unavailable and decisions are made in minutes on RA/PCWP, PAPI, and lactate or end-organ trajectory. The second is an elective multimodality tier for chronic TR and LVAD candidacy, where RV free-wall strain, coupling, contractile and afterload reserve testing (dobutamine or exercise RV–PA coupling) (89) and congestion staging can be integrated before committing to valve intervention, ventricular support, or a decision to defer or decline.

**Figure 4:**
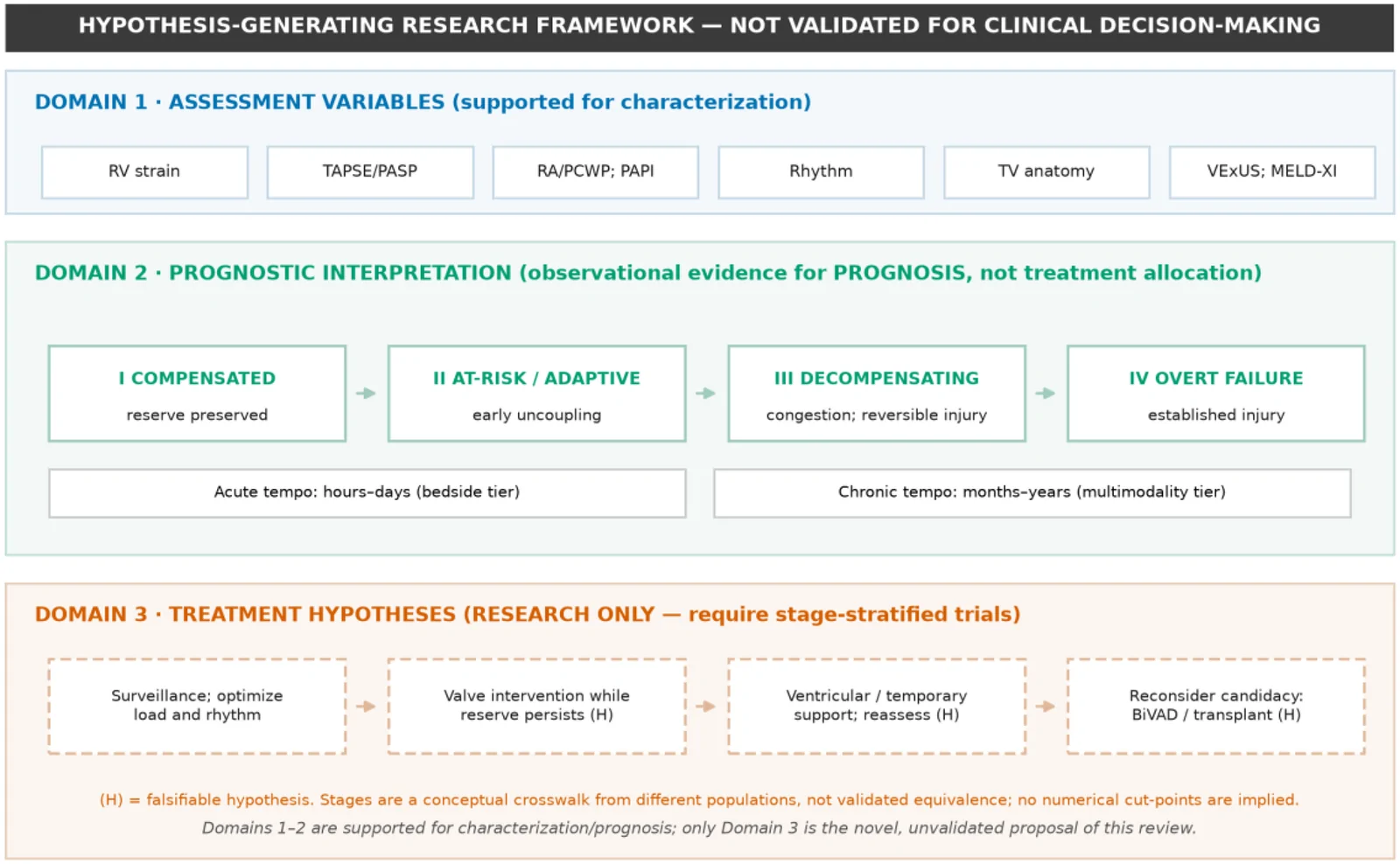
Proposed RV-first pathway across acute shock and durable LVAD candidacy, explicitly presented as a hypothesis-generating research framework, not a validated clinical decision algorithm. It separates three domains: (1) assessment variables supported for characterization; (2) prognostic staging supported by observational evidence for prognosis but not treatment allocation; and (3) research-only treatment hypotheses requiring stage-stratified validation. No numerical cut-points are implied, and dynamic multivariable reassessment is preferred to any single resting value.

Because RV performance is load-dependent, a single resting measurement is an unreliable basis for this judgment. Dynamic assessment should therefore combine serial echocardiography and invasive hemodynamics repeated after optimization—diuresis, afterload reduction, inotrope challenge and, in supported patients, controlled changes in LVAD pump speed—with trajectories of renal and hepatic function and of venous congestion (e.g., VExUS). Where feasible, pharmacological or exercise-based assessment of RV–pulmonary arterial coupling reserve can reveal reserve hidden at rest (89) although such testing is neither standardized nor always feasible in overt cardiogenic shock and should be regarded as investigational in that setting. A patient who moves toward a better stage after optimization differs prognostically from one who does not, and that response—rather than any isolated baseline number—is the more defensible timing signal (41, 42).

### 6.4 What the framework implies, and how it could be wrong

Reorganizing around the RV–tricuspid unit yields concrete direction on the field's three central controversies (Table 2). On concomitant tricuspid surgery at LVAD implantation, it predicts, consistent with observational registries (no randomized trial exists), that repair performed by reflex will not help, because it addresses the valve without changing the ventricle's trajectory. The testable corollary is that a staged, ventricular-reserve-guided approach should outperform anatomy-triggered repair (23, 24, 50). On transcatheter intervention, it predicts that selecting candidates by RV stage, and intervening earlier in that stage, might, if a stage-dependent effect exists, be necessary to convert the established symptom benefit into a survival benefit—an unproven hypothesis that must not be assumed (16, 84). On acute RV support, it predicts that outcome gains will come from earlier, stage-triggered deployment rather than from new devices alone (61).

**Table 2:** Three controversies reframed as RV-first, falsifiable predictions.

Controversy	Current evidence	Design/level of evidence	RV-first prediction (hypothesis)	Falsification condition	Relevant evidence
Concomitant tricuspid surgery at LVAD implantation	No consistent reduction in RVAD need or mortality and greater morbidity in observational STS-database and propensity-matched EUROMACS analyses (confounded by indication); the >2,200-patient MOMENTUM 3 portfolio analysis likewise found more RV failure, bleeding and stroke and no survival benefit (23, 24, 50).	Observational registries/PS-matched and trial-portfolio analyses; no dedicated RCT	Reflex, anatomy-triggered repair will not help; a staged, reserve-guided approach should outperform it	Reserve-guided staged repair fails to beat anatomy-triggered repair in severity-matched patients	No dedicated RCT; registries (23, 24, 50–54)
Symptom–survival dissociation after T-TEER/TTVR	TR and symptoms improved; pivotal RCTs met hierarchical composite endpoints (win ratios 1.48 and 2.02) driven substantially by quality-of-life/functional ranks; individual mortality and heart-failure-hospitalization components were not significant and trials were not powered for mortality; possible concentration of benefit in mid-range RV function is observational (15, 16, 74, 84). At ESC 2026 the randomized TRIC-I-HF trial in high-risk severe TR met a hierarchical primary (win ratio 2.42) and reduced three-year death/HFH (hazard ratio 0.40), the first randomized hard-event signal, but it did not stratify by RV stage (25, 83).	RCTs with hierarchical composite endpoints and limited follow-up, plus an observational subgroup signal	Hypothesis: if a stage-dependent effect exists, selecting/timing by RV stage may improve hard outcomes (unproven)	TR reduction improves survival equally regardless of RV stage (null treatment-by-stage interaction)	TRILUMINATE Pivotal, TRISCEND II (15, 16); TRIC-I-HF (25); none stage-stratified; TRIC-I-HF results (83)
Timing of acute RV support	Registry mortality >50% with late deployment (22). DanGer Shock (a left-sided Impella CP that excluded overt RV failure) reduced 180-day death from 59% to 46% (HR ≈0.74) but is not a test of dedicated RV support or RV-stage timing (2, 61).	One RCT of left-sided unloading in a selected population + observational RVAD registries; no RCT of stage-triggered RV support	Hypothesis: gains may come from earlier, stage-triggered deployment rather than from new devices alone	Earlier, stage-triggered support fails against later support in severity-matched patients	No dedicated RV-support RCT; observational (22, 61); left-unloading RCT (2)

This framework is intended to be falsifiable, and we state the conditions explicitly and quantitatively rather than rhetorically. The staging-primacy claim (Sections 6.1–6.2) fails if a randomized trial shows that transcatheter TR reduction improves survival to a similar degree regardless of baseline RV stage, that is, if the treatment-by-RV-stage interaction is null. The timing principle (Section 6.3) fails if earlier, stage-triggered RV support does not improve outcomes when tested against later support in patients matched on baseline severity. Until recently, no trial was designed to test the core prediction. TRIC-I-HF has now shown that a transcatheter repair strategy reduces death and heart-failure hospitalization in a high-risk severe-TR cohort (Section 5.1), but because it did not stratify or randomize by RV stage, it establishes that treating this population helps without isolating whether ventricular staging determines the magnitude of benefit (25, 83). Testing the framework properly will require trials that pre-specify RV-stage strata and randomize within them, precisely the design the proposed staging instrument (Table 3) is meant to enable. Stating the framework in these terms converts a set of disconnected observations into predictions that can be wrong, and it defines the trial architecture that would show whether they are.

**Table 3:** A conceptual crosswalk (a research hypothesis, not a validated unified stage) aligning descriptors of acute shock, durable-support RV failure and chronic tricuspid disease; no numerical cut-points are implied.

Crosswalk stage (descriptive)	Concept	Acute-shock descriptor (SCAI)	Durable-LVAD descriptor (MCS-ARC RVF)	Chronic-TR descriptor	Descriptive assessment variables (directional only; NO validated cut-points)	Research-only treatment hypothesis (NOT a validated trigger)
I Compensated	Coupled; reserve preserved	A–B	No RVF (pre-implant)	≤moderate TR; no congestion	Relatively preserved RVFWLS and TAPSE/PASP; low RA/PCWP; PAPI preserved; no venous congestion (VExUS 0)	H: surveillance; treat underlying substrate
II At-risk/adaptive	Dilating; coupling stressed	C	Risk factors present; no overt RVF	Severe TR; early congestion	Declining RVFWLS/TAPSE/PASP; rising RA/PCWP; intermediate PAPI; early venous congestion (VExUS 1)	H: optimize load/rhythm; valve intervention while reserve persists (hypothesis)
III Decompensating	Uncoupled; active congestion	D	Early acute/early post-implant RVF requiring support	Severe/torrential TR + reversible organ dysfunction	Low RVFWLS/TAPSE/PASP; high RA/PCWP; low PAPI; VExUS 2; rising MELD-XI	H: ventricular/temporary support ± valve; reassess; avoid isolated valve therapy (hypothesis)
IV Overt failure	Established multi-organ injury; reserve exhausted	E	Severe RVF/rescue RVAD; late RVF	End-stage congestive hepatopathy/nephropathy	Refractory hemodynamics; established end-organ failure despite optimization	H: reconsider candidacy; rescue only with realistic goals

RVFWLS = RV free-wall longitudinal strain. Rows map constructs developed in different populations for descriptive comparison only; they do not assert validated equivalence and must not be used as treatment triggers. Variables are directional rather than numerical thresholds: the commonly cited PAPI value (∼1.85) is cohort-dependent, TAPSE/PASP and RVFWLS must be interpreted relative to loading conditions and local reference ranges, and dynamic reassessment after optimization is preferred to any single resting value (see Section 6.2).

## 7 Conclusions and future directions

Across the mechanical circulatory support spectrum, from acute infarct-related shock to durable left ventricular assist device therapy, the right ventricle and its valve are important determinants of whether an intervention that succeeds mechanically also succeeds clinically. We have argued that the field's recurring disappointments are not independent problems but manifestations of one. They include the failure of concomitant tricuspid surgery to improve LVAD outcomes (23, 24) the dissociation between symptom and survival benefit after transcatheter tricuspid intervention (15, 16) and the poor results of right ventricular support deployed into established multi-organ failure (22). The shared root is intervening on a component of the RV–tricuspid unit without assessing, staging, or timing the intervention around the unit as a whole.

RV dysfunction, hemodynamic RV failure and systemic right-heart congestion are distinct entities and post-implant events should be reported with the time-bounded MCS-ARC definitions. RV–tricuspid coupling and congestion measures add prognostic information beyond ejection fraction; early RV failure after durable implantation and late rescue RV support carry high observed mortality. Contemporary randomized trials establish that transcatheter tricuspid repair and replacement reduce TR and improve symptoms, functional capacity and quality of life in selected patients. The device-pivotal TRILUMINATE Pivotal and TRISCEND II trials were not powered for—and did not demonstrate—a mortality benefit (15, 16, 74) whereas the higher-risk, strategy-based TRIC-I-HF trial reduced the composite of all-cause death or heart-failure hospitalization (with an unadjusted secondary survival signal), the first randomized evidence of hard-event reduction with tricuspid repair (83). Observational data consistently associate late, rescue interventions with worse outcomes than treatment while reserve is preserved.

The repeated observation that outcomes worsen with advancing RV–tricuspid stage across settings supports an integrated, dynamic RV-first assessment that combines coupling, congestion and organ-injury markers and is repeated after optimization; it is reasonable to infer that physiology-guided selection is preferable to selection by TR grade or anatomy alone, although this has not been established in randomized comparisons.

The central unproven proposition is that stage-triggered treatment improves hard outcomes. Testing it requires stage-stratified trials with endpoints matched to disease stage: mortality alone is often impractical and underpowered in early tricuspid disease, while symptom scores alone cannot establish disease modification. We favor hierarchical/composite and recurrent-event endpoints integrating survival, transplantation, durable or temporary RV support, heart-failure admissions, diuretic burden, renal/hepatic function, functional capacity and quality of life, residual TR and RV remodeling, and days alive and out of hospital, consistent with TVARC recommendations (85); LVAD studies should pre-specify MCS-ARC early acute, early post-implant and late RV failure (17) and shock studies should align with SHARC definitions (90). Recent guidelines move in this direction: the 2025 ESC/EACTS valvular guidelines recommend transcatheter tricuspid intervention (Class IIa, level A) in high-risk symptomatic severe TR on medical therapy only in the absence of severe RV dysfunction or pre-capillary pulmonary hypertension (91) and the 2025 ACC expert consensus decision pathway embeds RV and pulmonary-vascular assessment within tricuspid decision-making (92) supporting an RV-first approach while leaving its treatment-timing hypotheses to be tested. The 2026 TRIC-I-HF trial demonstrates that adequately powered, event-based tricuspid trials are feasible and that hierarchical endpoints can capture both symptom and hard-event benefit (83); stage-stratified designs remain necessary to determine in whom that benefit is greatest.

The RV-first framework we propose is intended less as a finished protocol than as a testable reorientation. Its three principles translate directly into predictions that can be confirmed or refuted: assess the unit rather than the valve, stage the ventricle rather than the regurgitation, and time intervention before the prognostic threshold rather than after. The most immediate priorities follow from the gaps this synthesis exposes. First, patient selection for transcatheter tricuspid therapy should be tested prospectively against measures of RV reserve rather than TR grade alone, using trial designs that stratify or randomize by RV stage. TRIC-I-HF has now established that treating a high-risk severe-TR population reduces events, yet because it did not stratify by RV stage it still cannot isolate ventricular staging as the operative variable (25, 83); future trials should randomize within pre-specified RV-stage strata (93, 94). Second, the timing of right ventricular support in acute shock requires dedicated randomized evaluation that separates the effect of earlier deployment from that of baseline severity, since current observational data cannot (2, 61). Third, the role of transcatheter tricuspid intervention in the LVAD population, untouched by randomized data, demands purpose-built study rather than extrapolation from surgical experience.

None of these questions can be answered by continuing to optimize the three tracks in isolation. A shared staging language, a common set of RV-centered endpoints, and trial designs that select and time by ventricular reserve would allow the fields to inform one another. Our framework may prove wrong in its specifics, which is precisely the point of stating it in falsifiable terms. Whether or not its particular thresholds survive testing, the evidence assembled here supports a focused proposition: treating the right ventricle as an organizing principle, rather than an afterthought to left-sided support and valve anatomy, is a hypothesis worth testing directly, and one that current practice has not yet put to the test.
